# Supplementary material for: Investigating the missing-wedge problem in small-angle X-ray scattering tensor tomography across real and reciprocal space
Source: J Synchrotron Radiat. 2024 Aug 28;31(Pt 5):1327–39. doi: 10.1107/S1600577524006702 (PMC11371061; doi:10.1107/S1600577524006702)
Supplement: Supplementary file 1 [file s-31-01327-sup1.pdf]

JOURNAL OF SYNCHROTRON RADIATION

Volume 31 (2024)

Supporting information for article:

Investigating the missing-wedge problem in small-angle X-ray scattering tensor tomography across real and reciprocal space

Leonard C. Nielsen, Torne Tänzer, Irene Rodriguez-Fernandez, Paul Erhart and Marianne Liebi

Supplementary Note S1. Real-space distribution of overall reciprocal-space errors

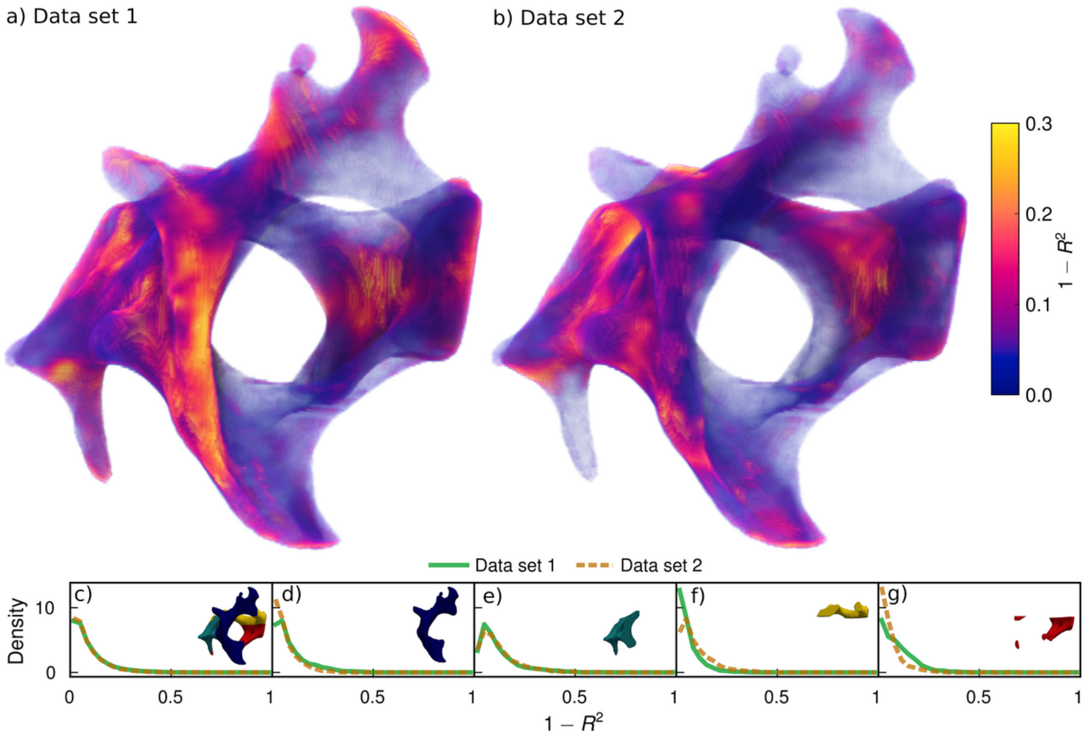

Fig. **S1**. **Volume render of reconstruction error.** The volume render shows the spatial distribution of reconstruction errors between a reconstruction from the full dataset and a reconstruction from **a)** data set 1 only, and **b)** data set 2 only. **c)**–**g)** show the distribution of errors in the whole sample, as well as across 4 partitions, with each partition being indicated by a colored inset. The R^2 in the error is the squared Pearson Correlation Coefficient for the reciprocal space map in each voxel. The error distribution is similar to the distribution of orientation errors, with the highest errors occurring in interface and edge regions.

Supplementary Figure **S1** shows the overall reciprocal space map error. Most of the errors appear to be in the flatter interface regions on the left- and right-hand side. The distributions of the reciprocal space map errors are largely similar to the distributions of the orientation errors. The distributions of errors across 4 partitions show that the better-performing data set varies depending on the local structure. Across the whole sample, panel c), the two data sets perform very similarly. The blue area, panel

d), has several protrusions which generally appear to perform better in data set 2, which therefore performs somewhat better overall. In the teal region, panel e), which is more blocky in its structure, the data sets perform approximately equally, but as is evident from the renders in panels a) and b), the errors are differently distributed. In the largely horizontally-oriented yellow part, panel f), data set 1 performs better, and the slightly more vertically-oriented red part, panel g), has data set 2 performing better. This shows the importance of considering the overall sample structure in order to optimize data acquisition. The error distributions are similar to the correlation coefficients obtained for simulated data in Nielsen *et al.* (2023).

Supplementary Note S2. Gaussian kernel representation

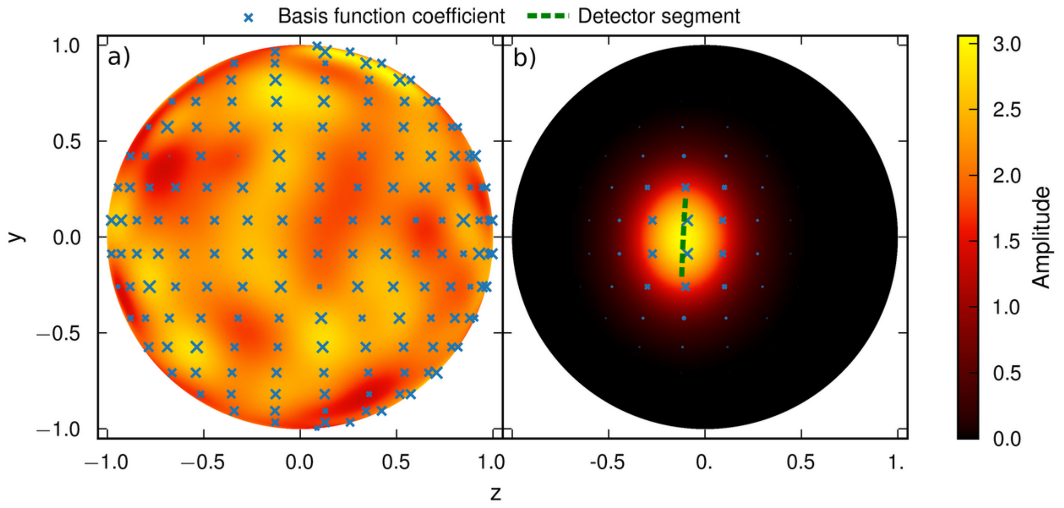

Fig. **S2**. **Gaussian kernel representations of spherical functions. a)** Spherical function with white noise coefficients. **b)** Mapping from Gaussian kernels to detector segment. The blue crosses show the location and magnitude of each coefficient. The green line shows the location of a detector segment on the sphere.

In Supplementary Figure **S2** we see two illustrations of the Gaussian kernel representation, including how the kernel functions are distributed using a modified Kurihara

mesh, what a function consisting of noise looks like, and how the detector segments (the arc of binned pixels on the detector over which the integral in Eq. (7) in the main text is carried out) map to the sphere (Kurihara, 1965). As the sample is rotated, a given detector segment is projected onto different parts of the reciprocal space sphere, per Eq. (3) in the main text. The principal modification of the Kurihara mesh consists in enforcing Friedel symmetry. In addition, the mesh is rearranged to make the distribution of points somewhat more regular. Moreover, the effect of the anisotropic distribution of points on the sphere is reduced by scaling each basis function by the value that is projected onto the point it is centered on in the grid when all coefficients are unity; this is the so-called "auto-projection" mentioned in the main work. Thus, basis functions centered on points which are in denser areas of the grid are scaled down, and functions centered on points in less dense areas of the grid are scaled up. At the equator, the Kurihara mesh contains only a semicircle of basis functions, since the representation respects Friedel symmetry. The number of points in the grid is decreased in each circle with increasing radius, to maintain approximately constant density of points. The standard deviation of the kernels is set based on the approximate distance between points to be wide enough to allow for the easy representation of unimodal distributions by multiple points. In addition, as can be seen in the b) panel, the width of the kernels implicitly enforce some of the continuity which is assumed of the reciprocal space map in the reconstruction.

Supplementary Note S3. Artefacts in data set 2

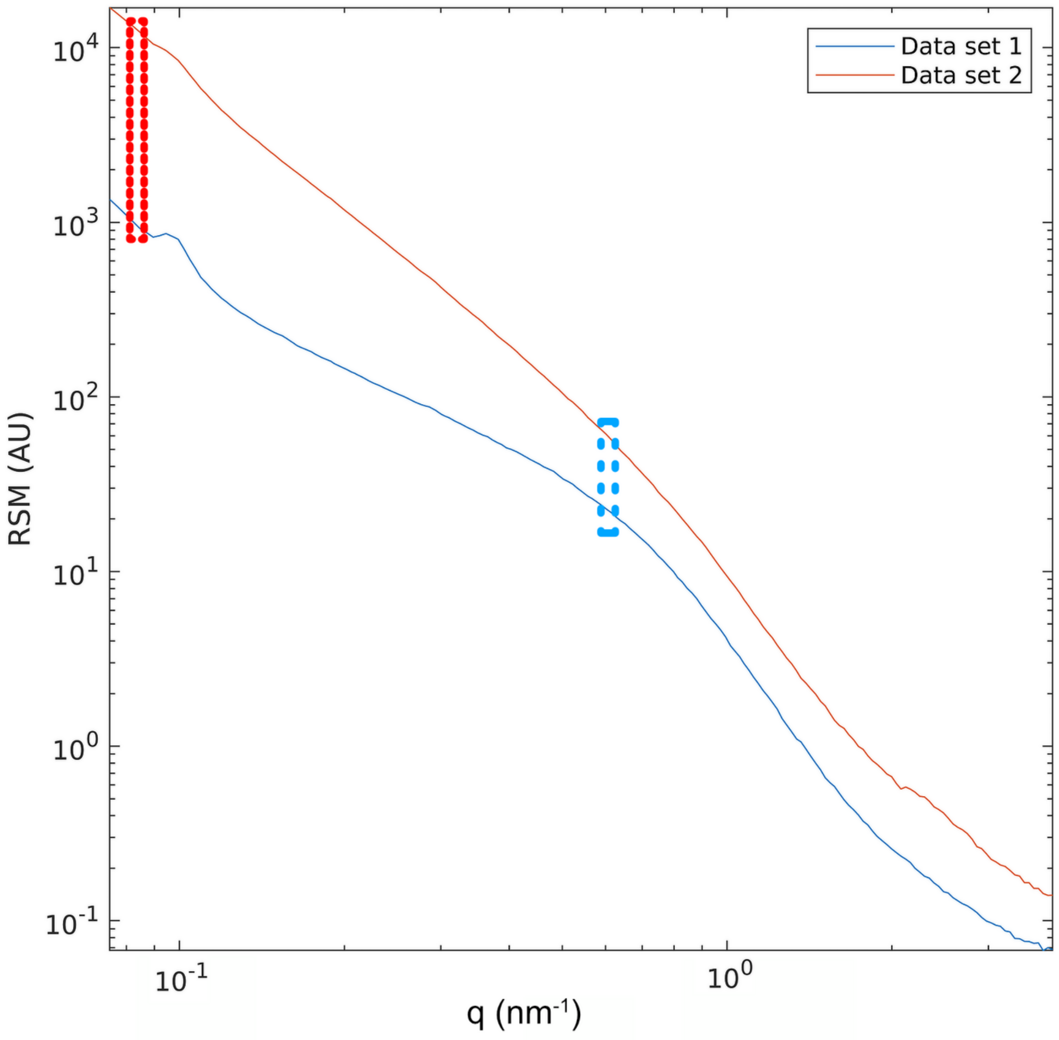

Fig. **S3**. **Scattering curves of data sets 1 and 2 for one pixel** Note the difference both in magnitude and shape of the scattering curve between the two data sets. The area enclosed by the red dashed outline indicate the q-region chosen for illustrating the large influence of the artefacts. The area enclosed by the blue outline indicates the q-region used for the reconstruction in the main work.

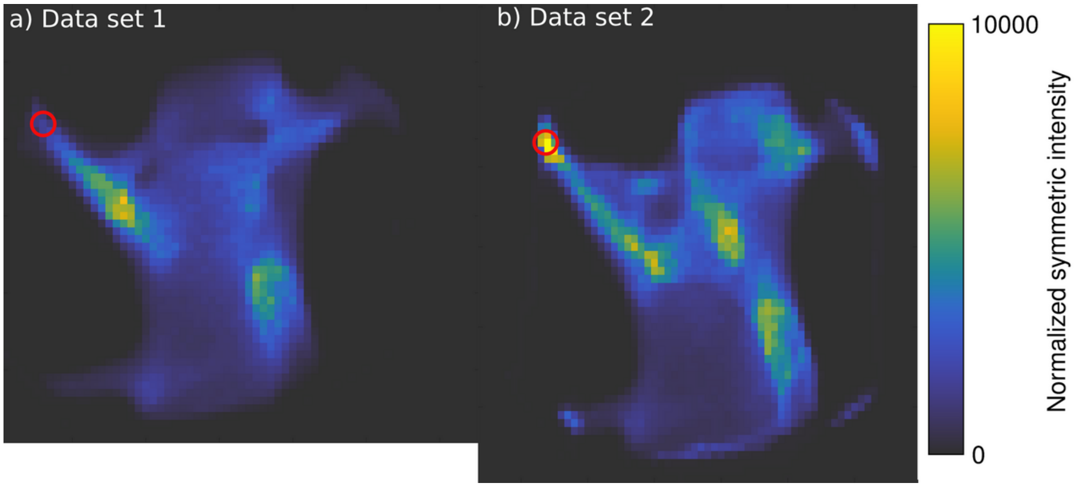

Fig. **S4**. **Raster scans from data sets 1 and 2 a)** Raster scan from data set 1. **b)** Raster scan from data set 2. The raster scans are colored by the transmission-normalized symmetric intensity around the q-value $0.0845\,\mathrm{nm}^{-1}$. The red circle indicate the pixel of the scattering curve shown in Supplementary Figure **S-3**.

In Supplementary Figure **S3** the azimuthally integrated scattering curve from a single measurement is shown for each of the data sets. The chosen points measure approximately the same location in the sample from approximately the same angle. The red outline highlights a high-q region shown in the raster scans in Supplementary Figure **S4**; the q-value for these scans is $0.0845\ \mathrm{nm}^{-1}$. The areas circled in red are the points where the scattering curves in Supplementary Figure **S3** were measured. As can be seen, the symmetric intensity is higher by an order of magnitude in data set 2, even though the views are very similar. The difference in the curves is much less around the higher q-range of $0.597\ \mathrm{nm}^{-1}$ to $0.607\ \mathrm{nm}^{-1}$, which is used in the main work and highlighted in green in Supplementary Figure **S3**, although still significant for the selected point.

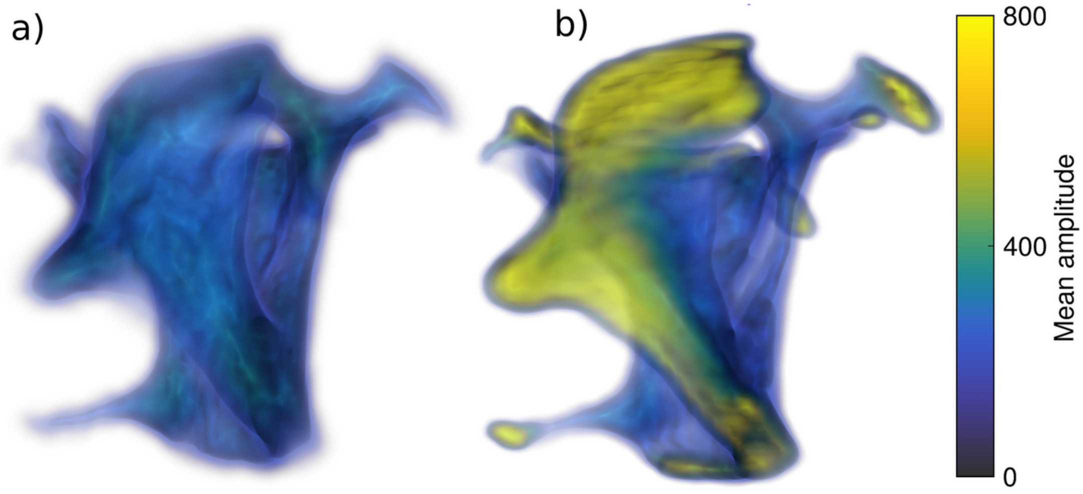

Fig. **S5**. **Volume renders of mean amplitude of low q-range reconstruction.** **a)** Volume render from data set 1. **b)** Volume render from data set 2. Note that the values above 800 are given zero opacity in order to render the figure clearly.

In Supplementary Figure **S5** we can see the differences in the mean amplitude (the reconstruction's counterpart to the normalized symmetric intensity) between the two data sets at this lower q-range in many regions, especially those close to the edges. The differences b oth i n t he r aster s can a nd i n t his r econstruction i s c onsistent with some artefact being introduced between the two measurements, with the most likely cause being the water-soluble glue mentioned in the main work.

Supplementary Note S4. Quantitative analysis of scalar quantities

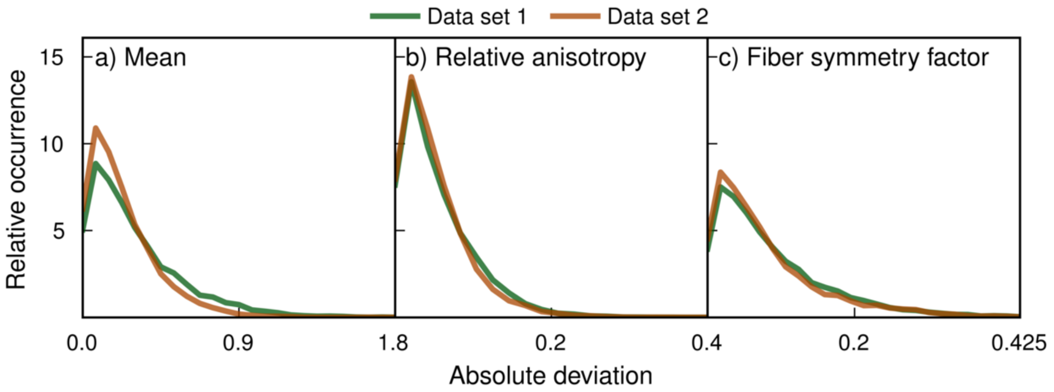

Fig. **S6**. **Absolute deviations of scalar quantities in sample. a)** Absolute deviation of mean. **b)** Absolute deviation of relative anisotropy. **c)** Absolute deviation of symmetry factor.

Frequency plots of the absolute deviations between the full reconstruction and each partial reconstruction for three scalar quantities are shown in Supplementary Figure **S6**.The limits for the plots are set based on histograms of the quantities. This shows the relative anisotropy as the most robust quantity. The mean is likely more robust than suggested here, due to artefacts introduced during re-mounting.

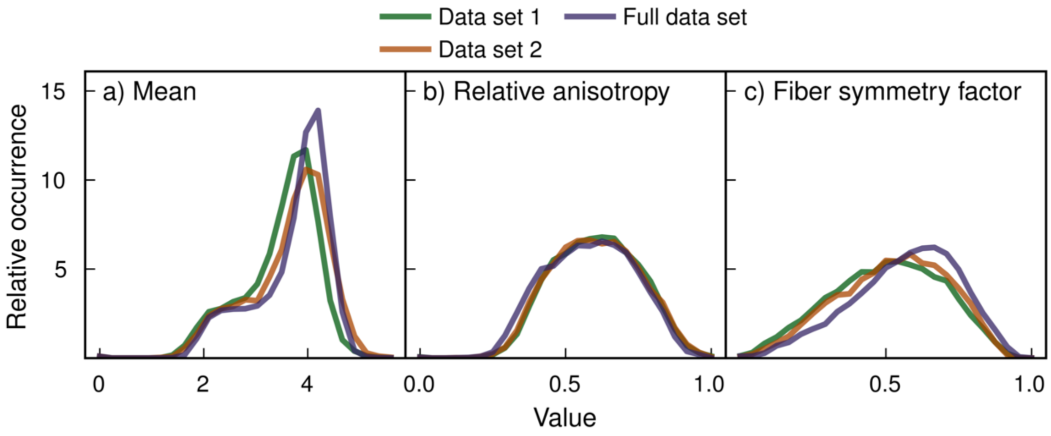

Fig. **S7**. **Relative occurrence of scalar quantities in sample. a)** Relative occurrence of mean. **b)** Relative occurrence of relative anisotropy. **c)** Relative occurrence of symmetry factor.

In Supplementary Figure **S7** we see the distribution of the three scalar quantities in the sample. It is clear from this plot that the mean of data set 2 is shifted in overall magnitude, which would be reflected in overall greater deviations in Supplementary Figure **S6** which reflect experimental artefacts, rather than theoretical robustness.

References

Kurihara, Y. (1965). *Monthly Weather Review*, **93**, 399–415.

Nielsen, L. C., Erhart, P., Guizar-Sicairos, M. & Liebi, M. (2023). Small-angle scattering tensor tomography algorithm for robust reconstruction of complex textures.
